# Youth depression in Ethiopia: a comprehensive systematic review and meta-analysis

**DOI:** 10.1186/s13034-025-00971-9

**Published:** 2025-10-22

**Authors:** Samuel Derbie Habtegiorgis, Animut Takele Telayneh, Lake Kumlachew, Nigussie Walelgn, Dawit Alemayehu, Molla Azmeraw, Kalkidan Worku Mitiku

**Affiliations:** 1 Department of Public Health, College of Health Sciences, Debre Markos University, Debre Markos, Ethiopia; 2 Department of Environmental Health Science, College of Medicine and Health Sciences, Debre Markos University, Debre Markos, Ethiopia; 3 Department of Surgery, Debre Markos University, Debre Markos, Ethiopia; 4 Department of Nursing, College of Health Science, Woldia University, Woldia, Ethiopia; 5 St. Peter’s TB Specialized Hospital, Addis Ababa, Ethiopia

**Keywords:** Youth, Depression, Ethiopia

## Abstract

**Background:**

Mental health is the state of well-being that manages our emotions, psychological stress, social well-being and it is essential at all stages of life. Depression is a mental health condition that causes repeated changes in mood and in how a person feels about everyday life. It can impact every aspect of life, including relations with friends, family, and the community. Depression, like most other mental health conditions, begins at the time of childhood or adolescence and can continue into adulthood. Depression among young people is widespread in developing nations like Ethiopia. This review aimed to compile data on Ethiopia’s youth depression.

**Methods:**

We searched through papers on the topic within the electronic databases of Worldwide Science, Google Scholar, Cochrane Library, PubMed, and Web of Science. The data were extracted using a Microsoft Excel™ format and exported to R (software) for management and further analysis. The heterogeneity among the former studies’ proportions was checked using the I2 test with p-values (I^2^ = 97, *p* < 0.01). Due to the presence of heterogeneity, a random-effects model was used to estimate the pooled depression among youth in Ethiopia. Additionally, subgroup analysis and publication bias were tested.

**Results:**

Overall pooled depression prevalence among youths in Ethiopia was 36% (95% CI: 31% to 42%). The level of social support, gender, and use of alcohol were significant predictors of youth depression.

**Conclusion:**

The magnitude of youth depression in Ethiopia is high as compared to the WHO mental health report (2022). Female gender, poor alcohol use, and a lack of social support were associated with youth depression. Educational and social support, particularly for females, and opportunities for youth to engage in various social and economic activities, should be implemented.

**Supplementary Information:**

The online version contains supplementary material available at 10.1186/s13034-025-00971-9.

## Introduction

Mental health is the state of well-being that manages our daily activities, including emotions, psychological stresses, social well-being or social interactions, and shapes our interactions, thinking, and how we view reality. Mental health is essential at all stages of life, from childhood, adolescence to maturity [1–3].

Depression is a mental health disorder that manifests itself with several repeated mood swings and feelings about day-to-day life. It affects nearly every part of life. It causes ongoing sadness, loss of interest, and difficulty thinking clearly. It can disturb sleep, appetite, and energy, while also leading to social withdrawal and strained relationships. At work or school, it reduces performance and increases absenteeism. If untreated, depression can contribute to chronic illness, economic hardship, and even suicide [6]. The onset of depression often occurs during childhood or adolescence. Depression is increasingly widespread in developing nations like Ethiopia [4, 5]. Like many diseases, depression is severe in some cases and mild in others. Depression can affect anyone, regardless of age, gender, location, income, social status, race, ethnicity, religion/spirituality, sexual orientation, background, or any other aspect of cultural identity. Although depression can develop at any age, three-quarters of cases begin before the age of 24.

 [7].

Depression affects approximately one in every five people at any given time [8]. In recent years, studies have shown that the prevalence of depression among adolescents is increasing [9]. Globally, over 300 million people are estimated to suffer from depression which is equivalent to 4.4% of the world’s population. From this about 17.5% are youth age less than 25 years. One in eight adolescents has a risk of depression problems [2, 10] and the prevalence of depression varies across the globe, in China (12.4%) [11], in America (20%) [12], in sub-Saharan Africa countries (18.3%) [13]. Suicide thoughts and attempts related to mental illness or depression has increased [14, 15] and every year more than 700,000 people die due to suicide around the world. It is the fourth leading cause of death among 15–29-year-olds and 77% of global suicides occur in low- and middle-income countries [16]. Ethiopia is experiencing an ongoing increase in the prevalence of depression particularly among young people [17]. It may be aggravated by the ongoing COVID-19 pandemic, war, and regional and national conflicts [18, 19]. Studies conducted about depression in Ethiopia show the different magnitudes and factors. The magnitude is different in different studies. This review aimed to generate pooled magnitude and risk factors of youth depression in Ethiopia.

## Methods

### Searching approach of primary studies

We searched through papers on the topic within the electronic databases of Worldwide Science, Google Scholar, Cochrane Library, PubMed, and Web of Science. Boolean terms were used to combine the keywords for the condition, context, and population to search: Consequently, Mental health illness*(tab) OR Mental health problem*(tiab) OR Mental Disorder*(tiab) OR Psychiatric Illness*(tiab) OR Psychiatric Disease*(tiab) OR Mental Illness*(tiab) OR Psychiatric Disorder*(tiab) OR Behavior Disorder*(tiab) OR Psychiatric Diagnosis*(tiab) OR Severe Mental Disorder*(tiab) OR depression(tiab) OR “Mental disorders“[MeSH Terms] AND Ethiopia(tiab) OR Federal republic of Ethiopia(tiab) OR “Ethiopia“[MeSH Terms] AND Adolescent*(tiab) OR Teen*(tiab) OR Teenager*(tiab) OR Youth*(tiab) OR School adolescent*(tiab) OR School age*(tiab) OR college student* (tiab) OR “adolescent“[MeSH Terms]. The literature was systematically searched in the specified database during the period from March 21, 2024, to April 27, 2024. The articles considered for this study were published between 2013 and 2024, up to the time of the search.

After the two authors (SDH & KWM) independently searched the original articles, any disagreements about the included articles were solved through dialogue and agreement in the presence of the third author (ATT). Using conventional Microsoft Excel sheets, ATT and SDH retrieved the data from the included papers and then transferred it to R statistical software for further analysis.

This systematic review and meta-analysis were presented according to the Preferred Reporting Items for Systematic Reviews and Meta-Analyses [20].

### Eligibility criteria

#### Inclusion criteria

We included studies for this review: (1) conducted on the mental health of high school, preparatory and college students (on adolescents and youth), (2) The outcome is mental health problems that include depression or depression disorder, and all types of observational studies conducted in Ethiopia, and published in English.

#### Exclusion criteria

Upon repeated requests from the cross-referring writers, articles with insufficient details were removed. Reviews and reports were not included in this study.

### Data extraction

We extracted all reported and this review objective-related data by using an independently prepared Microsoft Excel data extraction format. Data extraction format for the primary outcomes includes Authors, study period, publication year, study design, data collection tool, data analysis method, mean age of participants, study area-specific place where the study took place and region (according to Ethiopian political administration), sample size, response rate, and magnitude of depression.

For factors related to depression, the data extraction format was prepared for each specific variable separately in different sheets of Microsoft Excel. For each factor, we extracted the data in the form of two-by-two tables to calculate the odds ratio as an event with exposure or experiment with the event, total experiment with the event, event with control, and total with control. Any disagreements between the two authors during extractions were solved through discussion together with the third author.

### Outcome measurements

This meta-analysis and systematic review study has two outcomes: the primary outcome was to determine the prevalence, while the secondary outcome was to identify the factors associated with depression among youth. Concerning factors variables, we calculated the odds ratio from the primary studies using the two-by-two tables.

### Quality assessment

To assess the quality of the included cross-sectional studies, we used the Newcastle-Ottawa Scale tools [21]. The tool is divided into three main sections: (1) explores the study’s methodological quality; (2) reviews the studies’ comparability; and (3) evaluates the original publications’ quality of outcome measurement and statistical analysis.

### Statistical analysis

The data were extracted using a Microsoft Excel™ format, and the data were exported from Microsoft Excel to R (software) for management and further analysis. The data were presented using a text, table, and graph and forest plot. For this meta-analysis and review data with missing value were excluding from analysis. But before excluding it from the analysis different attempts have been implemented to find the missing data. Including contacting to principal investigator to request full data set. Furthermore, to exclude the data from analysis the situation of the missing data were considered. If the main outcome of this meta-analysis and review were not affected, included for final analysis.

### Evaluation of risk of bias

The heterogeneity among the proportion of the former studies was checked by using the I^2^ test together with the p-values (I^2^ = 97), *p* < 0.01). Due to the presence of significant heterogeneity between previous studies, a random-effects regression model was used to estimate the pooled depression among youth in Ethiopia. Additionally, subgroup analysis with some possible variables (region, sample mean variation and study participants) was complimented. To assess publication bias, a funnel plot and Egger test were used and it was asymmetry by visual inspection and the Egger’s test also showed the presence of publication bias (*p* < 0.0081).

## Results

### Primary articles search

**Fig. 1:**
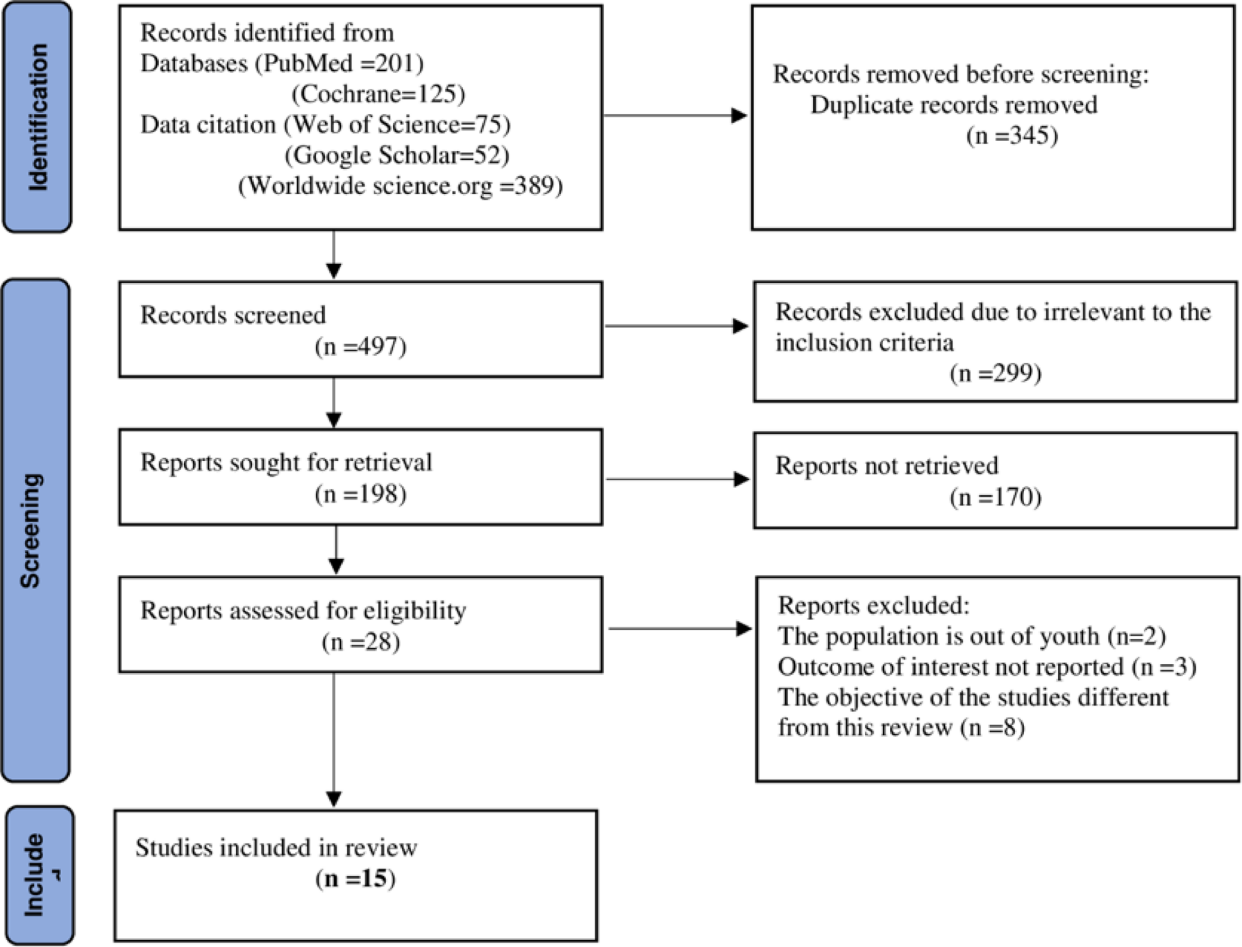
Showing flow diagram showing the process of selecting articles for meta-analysis youth depression in Ethiopia, 2024

From searched articles by using the earlier stated database and other repositories, 842 potential articles were identified and 345 were excluded due to duplication. By further reading the title of the article and abstracts from the remaining 497 articles, 299 articles were excluded due to irrelevant to the inclusion criteria. One hundred and ninety-eight articles were evaluated in whole text of the articles, then 174 articles were excluded for being irrelevant after careful reading. This systematic review and meta-analysis include only 15 articles that met the inclusion criteria (Fig. 1).

### Features of original articles

For these studies, 15 articles fulfil the inclusion criteria and are included for systematic review and meta-analysis. All included studies were conducted with cross-sectional study designs. Ten studies collected data using the Patient Health Questionnaire (PHQ-9/A) [22–27], and Depression, Anxiety, and Stress Scale-21 Items (DASS-21) [28–31] while the remaining studies used the Beck Depression Inventory (BDI-II) [32, 33], Kutcher Adolescent Depression Scale (KADS) [34], Center for Epidemiological Studies-Depression (CES-D) [35] and Hospital Anxiety and Depression Scale (HADS) [36] (Table 1).

**Table 1:** Included studies characteristics of depression in Ethiopia, 2024 (*n* = 15)

Authors	Study period	Data collection tools	Mean age	Area	Sample	Response rate %	Prevalence%
Chekol et al. [22]	June 18 to July 16, 2022	PHQ-9	18.15 ± 1.295	Bahir Dar city	584	96.9	22.1
Girma S et al. [23]	2, April to 30, May 2018.	PHQ-9	16.83 ± 1.3	Jimma town	561	97.3	28
Abebe et al. [32]	May and June 2016	BDI-II	18.6 ± 3.024	Addis Ababa	537	94.4	35.5
Demoze et al. [34]	May 01 to 30, 2016	KADS	N/A	Addis Ababa	465	97.4	36.4
Tirfeneh et al. [25]	January 1–30/2019	PHQ-9	N/A	Aksum	630	99.05	36.2
Abera et al. [28]	March 1 to 31, 2021	DASS-21	17.61 ± 1.83	Sawla	663	98.6	52.1
Gebreegziabher et al. [24]	June 8 -June 24, 2022	PHQ-9 A	N/A	Gondar town	1407	98	28.21
Nakie et al. [31]	Apr-2021	DASS-21	18.59 ± 1.792	North Gojjam	843	96.1	41.4
Tarecha et al. [26]	March to April 2021	PHQ-9 A	17.81 ± 1.47	Bahir Dar city	781	96.3	19.8
Gebremariam AT et al. [27]	3 July to 7 July 2020	PHQ-9	16.9 ± 1.813	Awash 7 kilo	392	96.2	28.91
Kebede et al. [36]	April to May 2017	HADS	1.61 ± 0.65	St. Paul Hospital	273	98.5	51.3
Hambisa et al. [37]	April 15 to 30, 2013	BDI-II	20.9 ± 2:17	HU	1040	98.3	26.8
Simegn et al. [29]	June 30 to July 30, 2020.	DASS-21	22.96	Gondar	423	100	46.3
Melaku et al. [30]	January 03 to 31, 2019	DASS-21	22.03 ± 2.62	Arsi	265	98.1	52.3
Lelisho et al. [35]	September 11 to 25, 2020	CES-D	N/A	MTU	779	100	39.5

### Data analysis and magnitude of depression

**Fig. 2:**
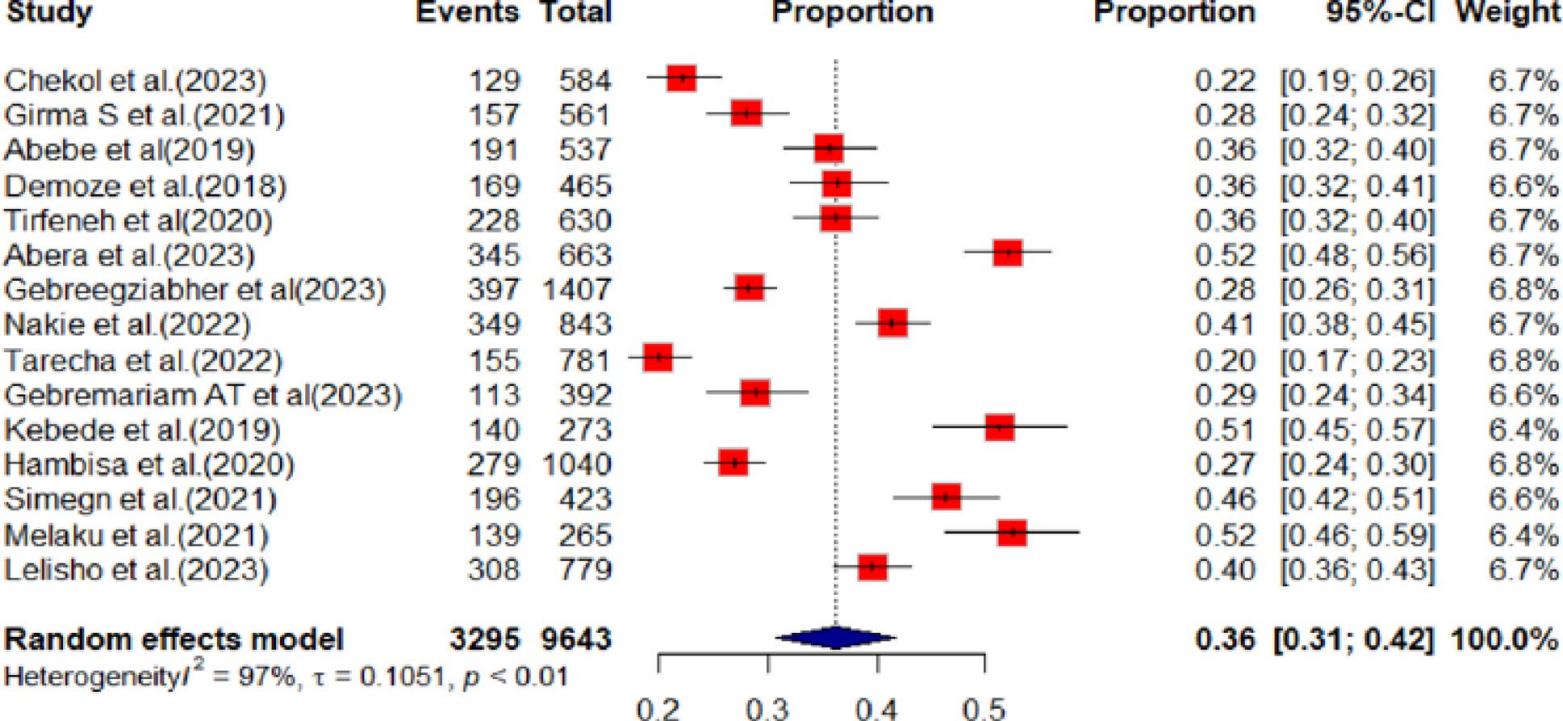
Showing the proportion of youth depression in Ethiopia, 2024

Overall depression prevalence among youths in Ethiopia was determined to be 36% (95% CI: 31% to 42%) (Fig. 2).

The random effect model was applied to estimate the depression among youth in Ethiopia, due to the significant existence of heterogeneity among primary studies (I^2^ = 97%, *p* < 0.01). To identify the potential sources of heterogeneity, we examined subgroup analysis with the region, sample means variation and study participants that could be likely characteristics related to the heterogeneity. Addis Ababa is the area where more depressed youth were found next to South Nations, Nationalities, and Peoples’ Regional State (SNNPRS)and Undergraduate university students are 10% more depressed than high school students (Table 2). To assess publication bias, a funnel plot was used and it was asymmetry by visual inspection which indicates the presence of publication bias. The Egger’s test also showed the presence of publication bias (*p* < 0.0081).

**Table 2:** Subgroup prevalence of youth depression in Ethiopia, 2024 (*n* = 15)

Variables	Characteristics	No, of studies	Total Participants	Prevalence (95% CI)
Region	Amhara	5	4038	31% (95% CI: 21;42)
	Oromia	2	826	40% (95% CI: 16;64)
	Addis Ababa	3	1275	41% (95% CI: 31;51)
	Tigray	1	630	36% (95% CI: 32;40)
	SNNSPRS	2	1442	46% (95% CI: 34;58)
	Afar	1	392	29% (95% CI: 24;34)
	Harari	1	1040	27% (95% CI: 24;30)
Sample mean	Lower	9	4130	37% (95% CI: 30;44)
	Higher	6	5513	35% (95% CI: 25;44)
Study participant	High school students	7	5300	33% (95% CI: 24;41)
	Undergraduate university students	5	2780	43% (95% CI: 34;53)
	HIV positive youth	1	537	36% (95% CI: 32;40)
	Orphan centred adolescents	1	465	36% (95% CI: 32;41)
	Community resident adolescents	1	561	28% (95% CI: 24;32)

### Factors associated with youth depression

Of the fifteen primary studies included in the review and meta-analysis, thirteen identified three variables that were significantly associated with depression.

**Fig. 3:**
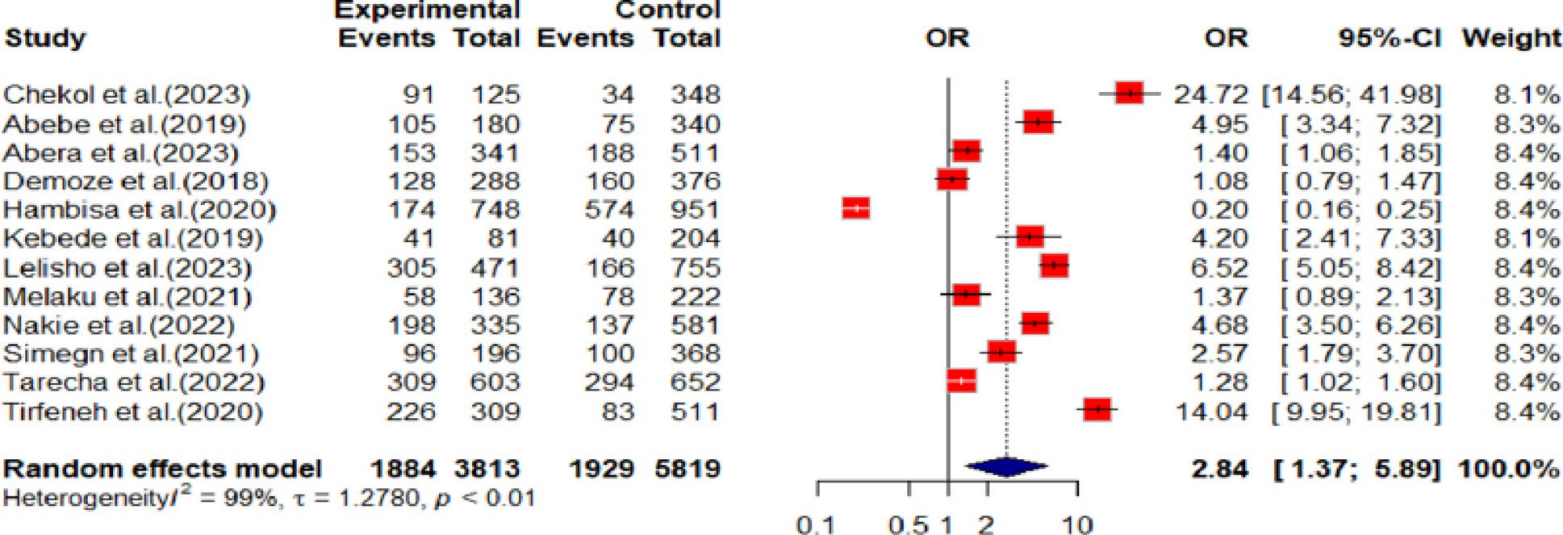
Showing sex difference and youth depression in Ethiopia,2024

Females were nearly three times more likely to be depressed (OR: 2.84, 95% CI: 1.37 to 6.89) as compared to male youth (Fig. 3).

**Fig. 4:**
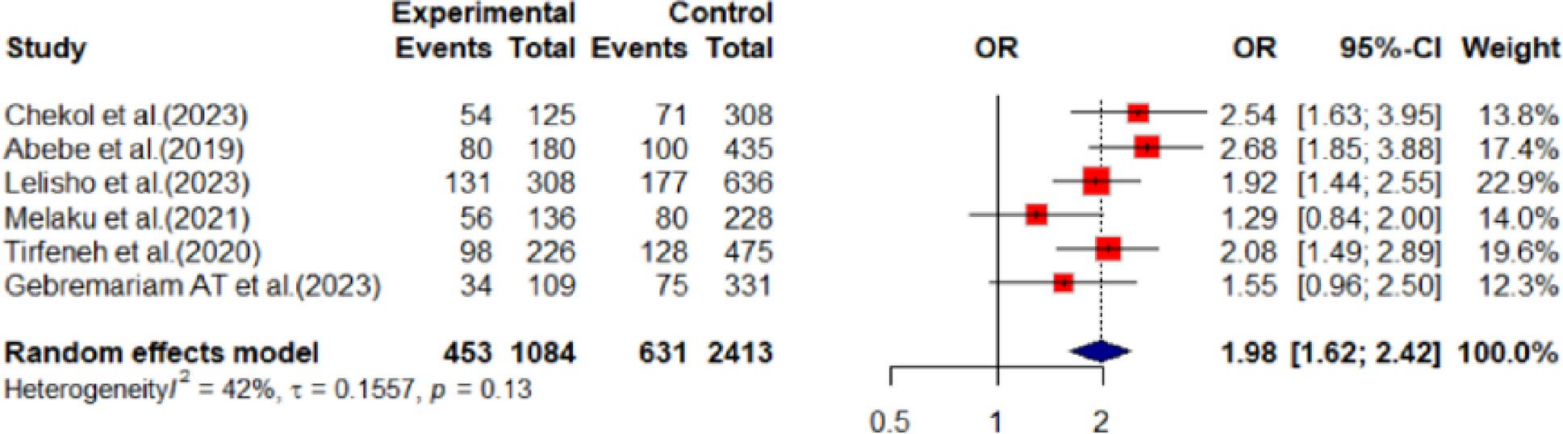
Showing ever alcohol use and youth depression in Ethiopia,2024

Youths who use alcohol are 98% more likely depressed (OR: 1.98, 95% CI: 1.62 to 2.42) when compared to the youths who didn’t ever use alcohol (Fig. 4).

**Fig. 5:**
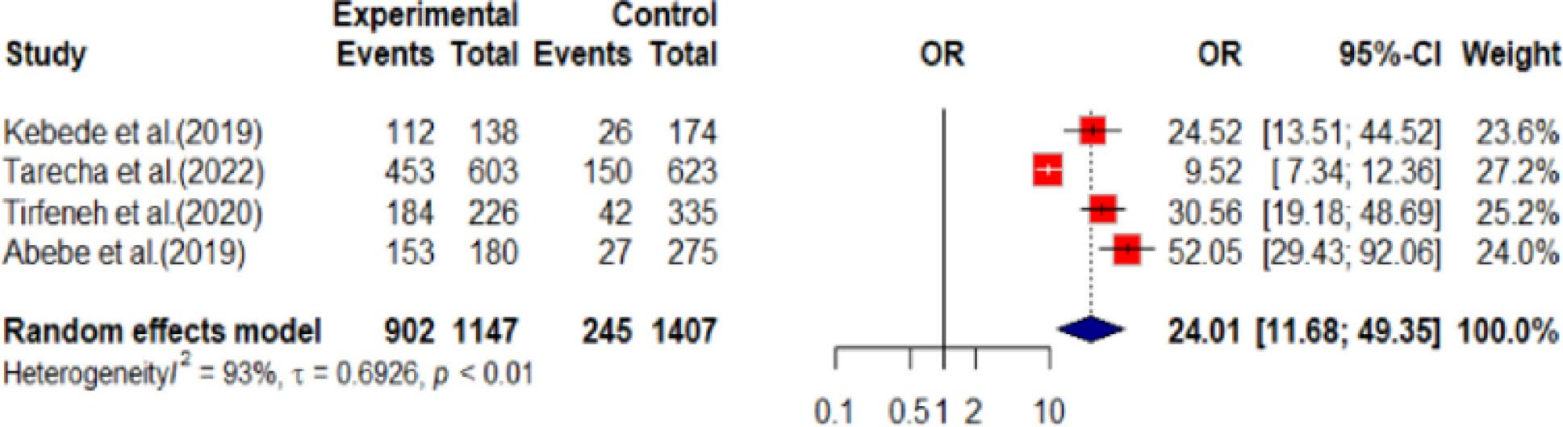
Showing social support and youth depression in Ethiopia,2024

Similarly, youths have 24 times the likelihood of not being depressed if they get social support (OR: 24.01, 95% CI: 11.68 to 49.35) compared to youths who lack social support (Fig. 5).

## Discussion

Depression is one of the most common mental disorders worldwide. Reported prevalence is higher in developed countries due to better detection, while the actual burden in developing countries may be equally high or even higher but often remains hidden because of limited resources and underdiagnosis. Half of all mental health disorders start before the age of fourteen tackling this issue on a global level is saving the later life burden of mental health disorders and will take its part in achieving SDGs3 [5, 38].

In this study, the overall pooled prevalence of youth depression in Ethiopia was 36% (95% CI: 31% to 42%), which is higher than systematic review and meta-analysis studies conducted in different countries [39], in the USA [40], and in Bangladesh [41]. Geographic differences may be the cause of the discrepancy, which is related to environmental aspects such as social stability, political stability, economic standing, and work availability. Instabilities in politics and the economy are prevalent across Ethiopia. However this study is aligned with a single study depression prevalence conducted in Meghalaya, India [42], global systemic review and meta-analysis [43], in Uganda [44] in South Africa [45].

But the prevalence of this study is lower than a study conducted in six Middle East countries [46], in Nepalese youth [47], and in Chandigarh, India [48]. The discrepancy may arise from the fact that the majority of research was carried out in the COVID-19 pandemic era. Around the world, the pandemic was annoying, and this could lead to a rise in youth depression prevalence.

This systematic review and meta-analysis also pooled the factors associated with the occurrence of depression among youths.

In this review, female youths were 2.84 times more likely to be depressed as compared to male youths. This study was supported by WHO reports [5], studies in Canada [49] and in Ethiopia [50].

This gap may be caused by a variety of circumstances, such as the possibility that female hormone fluctuations may cause depression [49], premenstrual issues, relationship issues, teenage pregnancy, abuse, a lack of social support, and similar circumstances. Similarly, youths who had ever used alcohol were more depressed as compared to non-alcohol users. This finding is also supported by different studies [42, 51–53]. The relationship between depression and alcohol use may be reciprocal. People with depression might drink regularly to cope with their symptoms, while even infrequent drinkers may experience depressive symptoms due to alcohol’s effects or withdrawal. Although drinking may provide short-term relief, over time it often worsens depressive symptoms and overall well-being.

This meta-analysis and systematic review indicate that social support plays a major role in young people’s depression. When compared to young people who receive adequate social support, those who receive insufficient social support are over 24 times more likely to be depressed. This finding is confirmed by various studies [54–56]. This may be related to the belief that a complex interplay of biological, psychological, and social components leads to depression. Adolescent sadness decreases when they receive an acknowledgement from the community and have got chance to participate in local issues. On the other hand, depression will be exacerbated as a youth is socially rejected, faces financial difficulty, instability in politics, economic crises, and other dysfunctional circumstances and conditions that worsen the depression of an individual’s life.

### Strengths and limitations of the study

The strength of this study is that it has tried to comprehensively review youth depression in Ethiopia. Additionally, the review addressed the main databases for this meta-analysis and systematic review, however, this systematic review and meta-analysis also have several limitations should be noted. Studies that were not fully accessible were excluded, potentially affecting the generalizability of the findings. Additionally, variations in the measurement tools used across the included studies may have influenced the pooled estimates of depression.

## Conclusion

This systematic review and meta-analysis revealed a substantially high prevalence of depression among youth in Ethiopia. Policymakers should acknowledge the significant impact of social support on mental health, given that inadequate support may result in severe consequences, and should implement strategies to enhance awareness and interventions at both community and family levels to effectively reduce the burden of youth depression.

## Supplementary Information

Below is the link to the electronic supplementary material.

Supplementary Material 1

## Data Availability

## The materials and data of this study are available from Samuel Derbie Habtegiorgis, the corresponding author upon request.
