# Supplementary material for: Youth depression in Ethiopia: a comprehensive systematic review and meta-analysis
Source: Child Adolesc Psychiatry Ment Health. 2025 Oct 22;19:115. doi: 10.1186/s13034-025-00971-9 (PMC12542379; doi:10.1186/s13034-025-00971-9)
Supplement: Supplementary file 1 — Supplementary Material 1 [file 13034_2025_971_MOESM1_ESM.zip › S1_Rcode..pdf]

Proportion

```
y<-metaprop(data=excel_depression, Event, Total, method="I",sm="PRAW", studlab = Authors, random
= T, common = F)


forest(
+     y,
+     common = FALSE,
+     random = TRUE,
+     overall = TRUE,
+     col.random = "black",
+     pooled.events = TRUE,
+     xlab = "Fig : proportion of youth depression",
+     smlab = "Proportion",
+     weight.study = "random",
+     col.study = "black",
+     col.square = "red",
+     col.diamond = "darkblue",
+     print.I2 = TRUE,
+     hetstat = TRUE,
+     hetlab = "Heterogeneity",
+     resid.hetstat = TRUE,
+     print.tau = TRUE,
+     fs.xlab = 14
+ )
```

OR

```
y<-metabin(data=excel_depression, Event.e,Total.e, Event.c, Total.c, method="Inverse",sm="OR", st
udlab = Authors, random = T, common = F)


forest(
+     y,
+     common = FALSE,
+     random = TRUE,
+     overall = TRUE,
+     col.random = "black",
+     pooled.events = TRUE,
+     xlab = "Fig 4: Showing ever alcohol use and youth depression", #Edited according to our variables
+     smlab = "OR",
+     weight.study = "random",
+     col.study = "black",
+     col.square = "red",
+     col.diamond = "darkBlue",
+     print.I2 = TRUE,
+     hetstat = TRUE,
+     hetlab = "Heterogeneity",
+     resid.hetstat = TRUE,
+     print.tau = TRUE,
+     fs.xlab = 14
+ )
```
