# Supplementary material for: Youth depression in Ethiopia: a comprehensive systematic review and meta-analysis
Source: Child Adolesc Psychiatry Ment Health. 2025 Oct 22;19:115. doi: 10.1186/s13034-025-00971-9 (PMC12542379; doi:10.1186/s13034-025-00971-9)
Supplement: Supplementary file 1 — Supplementary Material 1 [file 13034_2025_971_MOESM1_ESM.zip › S4_Table_3, bias and quality assement.docx]

Table showing the completed risk of bias and quality assessments for each study.

| Study | Risk of Bias (High/Low) | Quality Assessment (High/Moderate/Low) | Comments |
| --- | --- | --- | --- |
| Chekol et al | Low | Moderate | Validated tools (PHQ-9), but limited by the inability to draw causal conclusions. Use of appropriate statistical methods. |
| Girma S et al. | Low | Moderate | Robust tools (PHQ-9, Oslo scale), but limited by self-reporting and lack of causal inference. |
| Abebe et al. | Low | Moderate | Robust sample size and validated tools, but limited by inability to establish causality. |
| Demoze et al. | Low | Moderate | Robust sample size and validated tools, but limited by the inability to infer causality. |
| Tirfeneh et al. | Low | Moderate | High response rate and solid statistical methods, but limited by inability to establish causality. |
| Abera et al. | Low | Moderate | Large sample size, and a validated tool (DASS-21), but the limited causal conclusions. |
| Gebreegziabher et al. | Low | Moderate | Social phobia are significant risk factors. |
| Nakie et al. | Low | Moderate | Social phobia are significant risk factors. |
| Tarecha et al. | Low | High | Use of a validated tool improves reliability.  Robust statistical analysis. |
| Gebremariam AT et al. | Low | High | Standardized tools reduce variability and increase reliability. |
| Kebede et al. | Low | High | Standardized tools reduce variability and increase reliability. |
| Hambisa et al. | Low | High | The BDI is a validated tool for measuring depressive symptoms. |
| Simegn et al. | Low | High | DASS-21 is a validated, reliable tool for measuring mental health. |
| Melaku et al. | Low | Moderate | Self-reports may introduce response bias, particularly for sensitive behaviors. |
| Lelisho et al. | Low | Moderate | Self-reports may introduce response bias |
